# Dual-color FISH analyses of xenogeneic human fibroblast sheets transplanted to repair lung pleural defects in an immunocompromised rat model

**DOI:** 10.1186/s13104-024-06792-x

**Published:** 2024-05-15

**Authors:** Masato Kanzaki, Ryo Takagi, Shota Mitsuboshi, Hiroaki Shidei, Tamami Isaka, Masayuki Yamato

**Affiliations:** 1 Department of Thoracic Surgery, Tokyo Women’s Medical University, 8-1 Kawada-cho, Shinjuku-ku, Tokyo, 162-8666 Japan; 2 Institute of Advanced Biomedical Engineering and Science, Tokyo Women’s Medical University, TWIns, 8-1 Kawada-cho, Shinjuku-ku, Tokyo, 162-8666 Japan

**Keywords:** Fibroblast, Extracellular matrix, Pleural defect, Cell sheet, Temperature-responsive culture dish

## Abstract

**Background:**

Pulmonary air leaks (PALs) due to visceral pleura injury during surgery is frequently observed after pulmonary resections and the complication is difficult to avoid in thoracic surgery. The development of postoperative PALs is the most common cause of prolonged hospitalization. Previously, we reported that PALs sealants using autologous dermal fibroblast sheets (DFSs) harvested from temperature-responsive culture dishes successfully closed intraoperative PALs during lung resection.

**Objective:**

In this study, we investigated the fate of human DFSs xenogenetically transplanted onto lung surfaces to seal PALs of immunocompromised rat. Dual-color FISH analyses of human fibroblast was employed to detect transplantation human cells on the lung surface.

**Results:**

One month after transplantation, FISH analyses revealed that transplanted human fibroblasts still composed a sheet-structure, and histology also showed that beneath the sheet’s angiogenesis migrating into the sheets was observed from the recipient tissues. FISH analyses revealed that even at 3 months after transplantation, the transplanted human fibroblasts still remained in the sheet. Dual-color FISH analyses of the transplanted human fibroblasts were sparsely present as a result of the cells reaching the end of their lifespan, the cells producing extracellular matrix, and remained inside the cell sheet and did not invade the lungs of the host.

**Conclusions:**

DFS-transplanted human fibroblasts showed that they are retained within cell sheets and do not invade the lungs of the host.

## Introduction

Fibroblasts are the most common cells of connective tissues, providing repair and regenerative processes in human tissue and organ, becoming rapidly activated upon tissue damage to proliferate and migrate to damaged sites, and secreting extracellular matrix (ECM) components (e.g. collagens) that provide provisional scaffolds for normal repair events such as epithelial cell migration. Through ECM remodeling, fibroblasts provide mechanical strength [1]. Fibroblasts are important for maintaining homeostasis, directing regeneration, and controlling inflammation [2]. Furthermore, fibroblasts are also involved in immune surveillance, cancer progression, maintaining the stem cell niche, and pathologic fibrosis [3]. In brief, fibroblasts are known to be involved in the development of pulmonary fibrosis [4, 5]. In pulmonary fibrosis, the alveoli are repeatedly damaged for extended periods of time, and due to an excessive repair reaction, fibroblasts secrete excessive collagen thickening and hardening the alveoli walls [4, 5].

Previously, we described use of autologous dermal fibroblast sheets (DFSs) fabricated from temperature-responsive culture for pulmonary air leaks (PALs) sealants to successfully close intraoperative PALs during lung resection [6–8]. The use of fibroblast as a cell source for pleural substitutes cannot be ruled out as causing pulmonary fibrosis.

In the present study, we investigated whether fibroblasts engrafted after transplantation enter the lungs of the host and have extended this approach using human DFSs xenogenetically transplanted onto lung surfaces to seal PALs in immunodeficient athymic rats. Dual-color Fluorescence in situ hybridization (FISH) analyses of human fibroblasts and rat host cells were employed to distinguish transplanted human cells on the lung surfaces.

## Methods

### Human volunteer collection

Skin samples were surgically collected from a total of 4 volunteers who underwent surgery in the Department of Thoracic Surgery, Tokyo Women’s Medical University (Japan). These volunteers consisted of two men and two women, ranging in age from 76 to 87 years (median: 81 years). The clinical diagnosis of these volunteers was primary lung cancer in 2 persons, metastatic lung tumor in 1 person, and pneumothorax in 1 person.

### Culture of human dermal fibroblasts

These volunteers were each intubated with a double-lumen endotracheal tube and were placed in the lateral decubitus position under general anesthesia. A skin specimen of approximately 1 cm^2^ was resected from the surgical incision. As we previously reported, the skin specimen was transferred into a 50-mL centrifuge tube with Dulbecco’s modified Eagle’s medium (DMEM, Merck, Darmstadt, Germany) containing ampicillin sodium and sulbactam sodium (Unasin-S, Pfizer, NYC, USA), streptomycin sulfate (Meiji Seika Pharma, Tokyo, Japan), and amphotericin B (Fungizone, Bristol-Myers Squibb, New York, USA), and transported to biosafety cabinet [4–7]. The skin samples were cut into 3 mm^2^ pieces and treated with DMEM containing 1000 units/ml dispase (Godo Shusei, Tokyo, Japan) overnight at 4 °C. After all layers of epithelium were removed with forceps, remaining dermis was then minced, and subjected to collagenase dissociation. Dermal fibroblasts were plated at an initial density of 2 × 10^5^ cells/cm^2^ on 60 mm diameter culture dishes (BD Biosciences, Franklin Lakes, USA) and cultured for 3 days at 37 °C in a humidified atmosphere of 5% CO_2_ and the culture media was changed every 2 days. At 3 weeks, cells were harvested by treatment with 0.05% trypsin-ethylenediamine tetraacetic acid (EDTA) (Sigma, St. Louis, Missouri, USA) for 5 min at 37 °C. For subculture, harvested cell suspensions were seeded on temperature-responsive culture dish (UpCell™, CellSeed, Tokyo, Japan) at an initial density of 2 × 10^5^ cells/cm^2^ and then cultured for an additional 7 days. Dermal fibroblast sheets (DFS) were harvested by reducing cell culture temperature to 20 °C, prompting contiguous monolayer sheets to release from culture plastic and float in culture media. Sheets were recovered for transplant use by a non-stick polypropylene support sheet.

### Transplantation of human DFS to rodent surgical pleural defects

For DFS implantation, avoiding rejection by the rat’s immune system, six male F-344 athymic rats were anesthetized with 2% inhaled isoflurane and ventilated using a rodent ventilator. Under aseptic conditions, the animals were positioned in the right lateral decubitus position and a left-lateral thoracotomy in the fifth intercostal space was performed. A 5 mm long incision with a depth of 3 mm, was set in the left lung, using sterile surgical scissors. The resulting pleural defect was then covered by transplantation of harvested human DFS and held in place for several minutes, DFS spontaneous adhesion to the surgical site via ECM observed the need for any suturing. Five minutes after covering, no bleeding was observed from the injured lung, so the pleural space was sutured, closed, dermal layers also closed, and surgery was completed.

A re-left-lateral thoracotomy in the fifth intercostal space was performed under anesthesia in 4 out of 6 transplanted rats after 4 weeks and in the remaining 2 transplanted rats after 3 months, as previously described [4–6]. The transplanted area was directly visually observed and the left lung including the surgically operated site was subsequently resected for further analysis. Animals were finally euthanized by overdoses of pentobarbital.

### Histological analysis

Tissue were fixed with 10% formalin and routinely processed into 10 μm-thick paraffin wax-embedded sections. Hematoxylin and eosin (HE) and Azan staining were performed by conventional methods. Stained slices were observed under microscopy.

### Fluorescence in situ hybridization (FISH)

Dual-color FISH analysis was performed on formalin-fixed paraffin-embedded tissue sections. Briefly, sections of 5-µm thickness were deparaffinised, dehydrated, digested in pepsin solutionn (0.1% in 0.1 N HCl) at 37ºC for 1 min and dehydrated. Human and Rat Cot-1 DNA were labelled with Cy3 and digoxigenin respectively by nick-translation to generate species-specific FISH probes for human and rat. These labelled probes were mixed with sonicated salmon sperm DNA in hybridization solution. The probes were applied to the pretreated sections, covered with cover slips and simultaneously denatured at 80ºC for 10 min. Hybridization was carried out at 37ºC overnight. Sections were then washed with 50% formamide /2xSSC at 37 ºC for 20 min, 1xSSC for 15 min at RT, blocked by blocking solution (5% skim milk, 0.1% nonidet P-40, 0.1 M phosphate buffer pH7.5) at 37 ºC for 30 min. Detection of the digoxigenin-labelled probes was performed with Cy5-labeled anti-digoxigenin antibody. The slides were treated with antibodies at 37ºC for 30 min, washed 3 times with 0.1% Nonidet P-40/2xSSC, counter-stained by 4,6-diamidino-2-phenylindole (DAPI) and mounted. The FISH images were captured with the CW4000 FISH application program (Leica Microsystems Imaging Solution Ltd.) using a cooled charge coupled device (CCD) camera mounted on a Leica DMRA2 microscope.

## Results

### Fabrication of transplantable human fibroblast sheets (DFS)

**Fig. 1:**
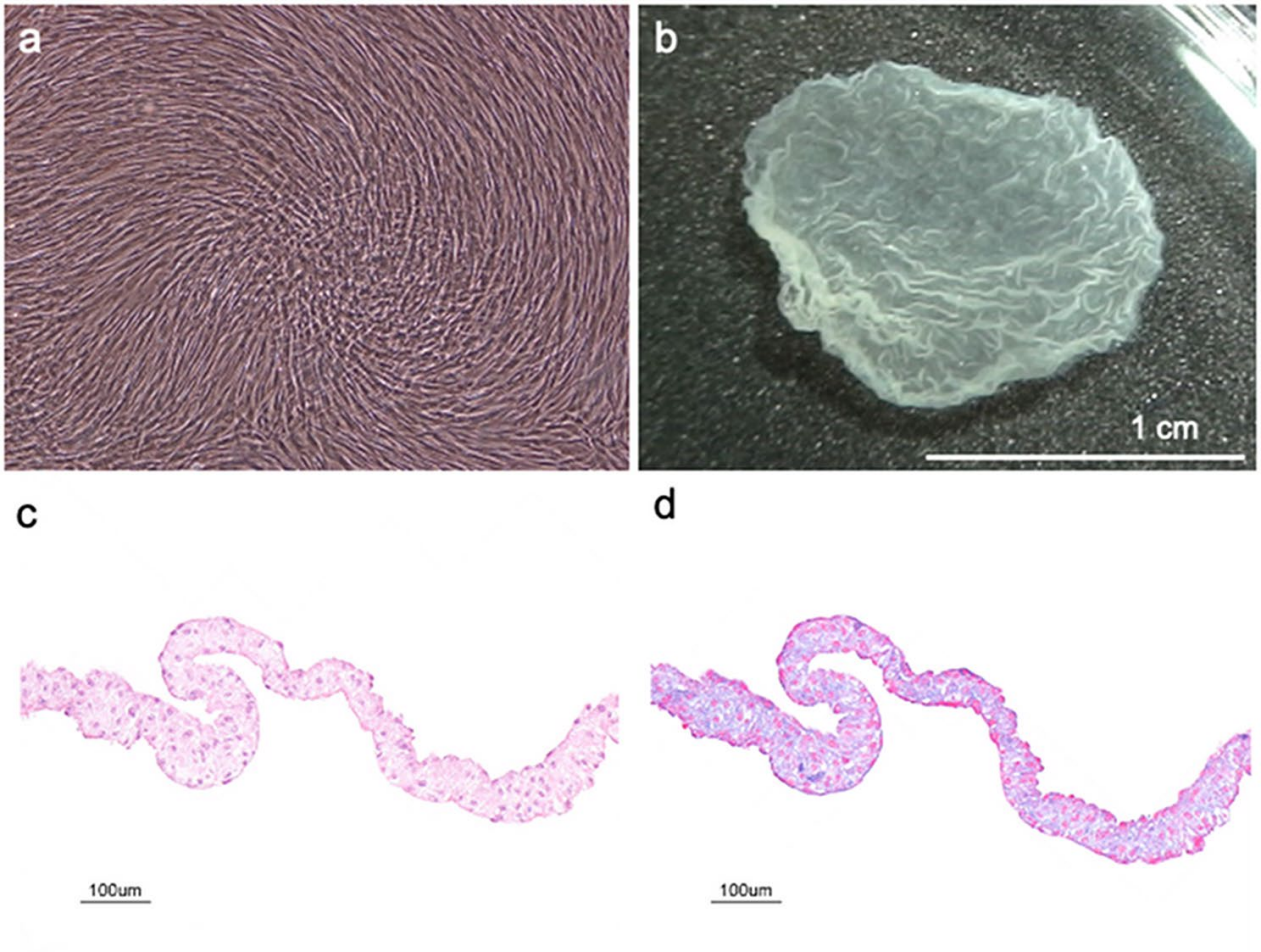
Fabrication of transplantable human dermal fibroblast sheets (DFSs). (**a**). Phase-contrast micrographs of confluently cultured human dermal fibroblasts on temperature-responsive culture dish before temperature reduction for cell sheet harvest. (**b**). Macroscopic view of a harvested DFS floating in culture medium after temperature reduction for cell sheet harvest. (**c**). Hematoxylin and eosin (HE) staining of a harvested DFS. No cell damage or defects of cell sheet was observed. (**d**). Azan staining of a harvested DFS. DFS comprises one to four stratified cell-dense layers stained red as well as abundant deposited extracellular matrix stained blue

Transplantable DFSs, resulted from proliferated human fibroblasts reaching confluency on 35-mm temperature-responsive culture dishes (CellSeed. Inc. UpCell™). Transplantable contiguous DFSs (Fig. 1a and b) consisted of one to four stratified cell-dense layers with abundant extracellular matrix present (Fig. 1c and d).

### DFS transplantation to rat

**Fig. 2:**
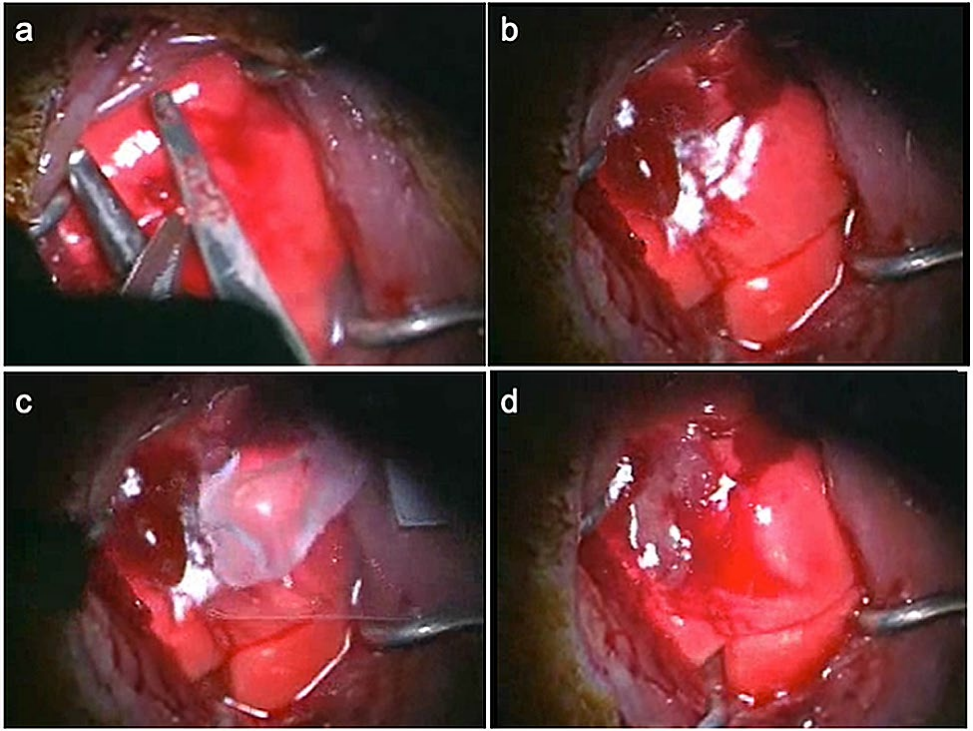
Transplantation of human dermal fibroblast sheets (DFSs) onto rat pleural defects. (**a**). A 5-mm-long incision with a depth of 3-mm was made by scissors after performing left-lateral thoracotomy in the fifth intercostal space. (**b**). Air-leakage and bleeding from the lung was confirmed. (**c**). The harvested human DFS was transferred from a non-adhesive polypropylene support sheet and transplanted to cover the pleural defect. (**d**). Five minutes after placing the DFS, there was no bleeding from the injured lung, the pleural defect was closed

A 5-mm-long incision with a depth of 3-mm was made by scissors and air leakage and bleeding from the lung was confirmed (Fig. 2a and b). The harvested human DFS was transferred from a non-adhesive polypropylene support sheet and transplanted to cover the pleural defect. (Fig. 2c). No bleeding from the injured lung was observed after 5 min, the pleural defect was closed and the surgery was completed. (Fig. 2d).

### Rat re-thoracotomy 4 weeks and 3 months after human DFS transplantation

**Fig. 3:**
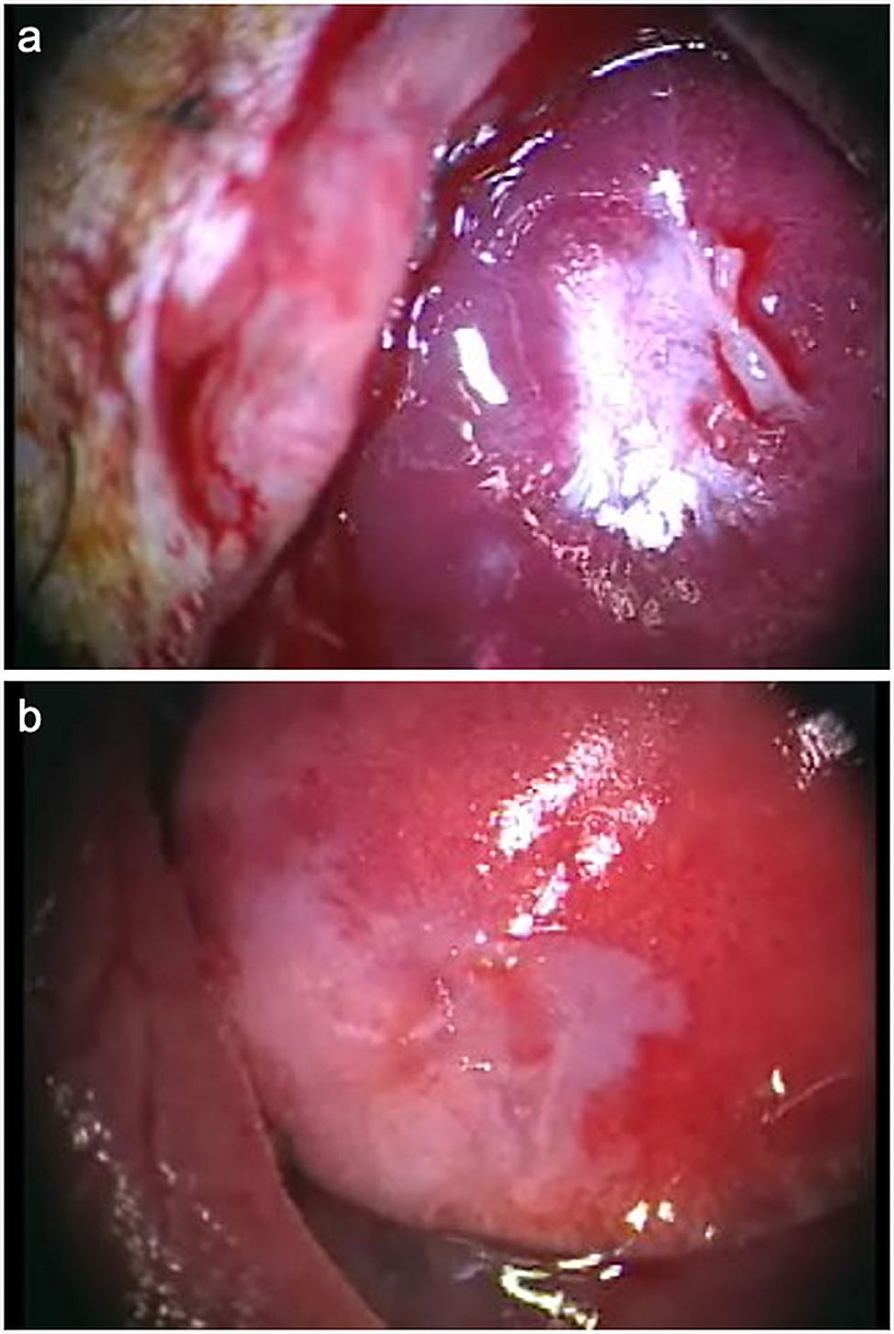
Re-thoracotomy after transplantation of human dermal fibroblast sheet (DFS). (**a**). Re-thoracotomy 4 weeks after DFS transplantation. The transplanted human DFS appears white on the lung surface and remains covering the pleural defect. (**b**). Three months after DFS transplantation, the transplanted human DFS still appear white on the lung surface, but contract in size. Blood vessels are observed penetrating into the DFS from host tissue

At both four weeks and 3 months after DFS transplantation, site of human DFSs had maintained a white visual DFS and covered the pleural defects in all cases (Fig. 3a and b). This site was harvested for analysis.

### Histology and FISH analyses of performance of transplanted human DFS sites

**Fig. 4:**
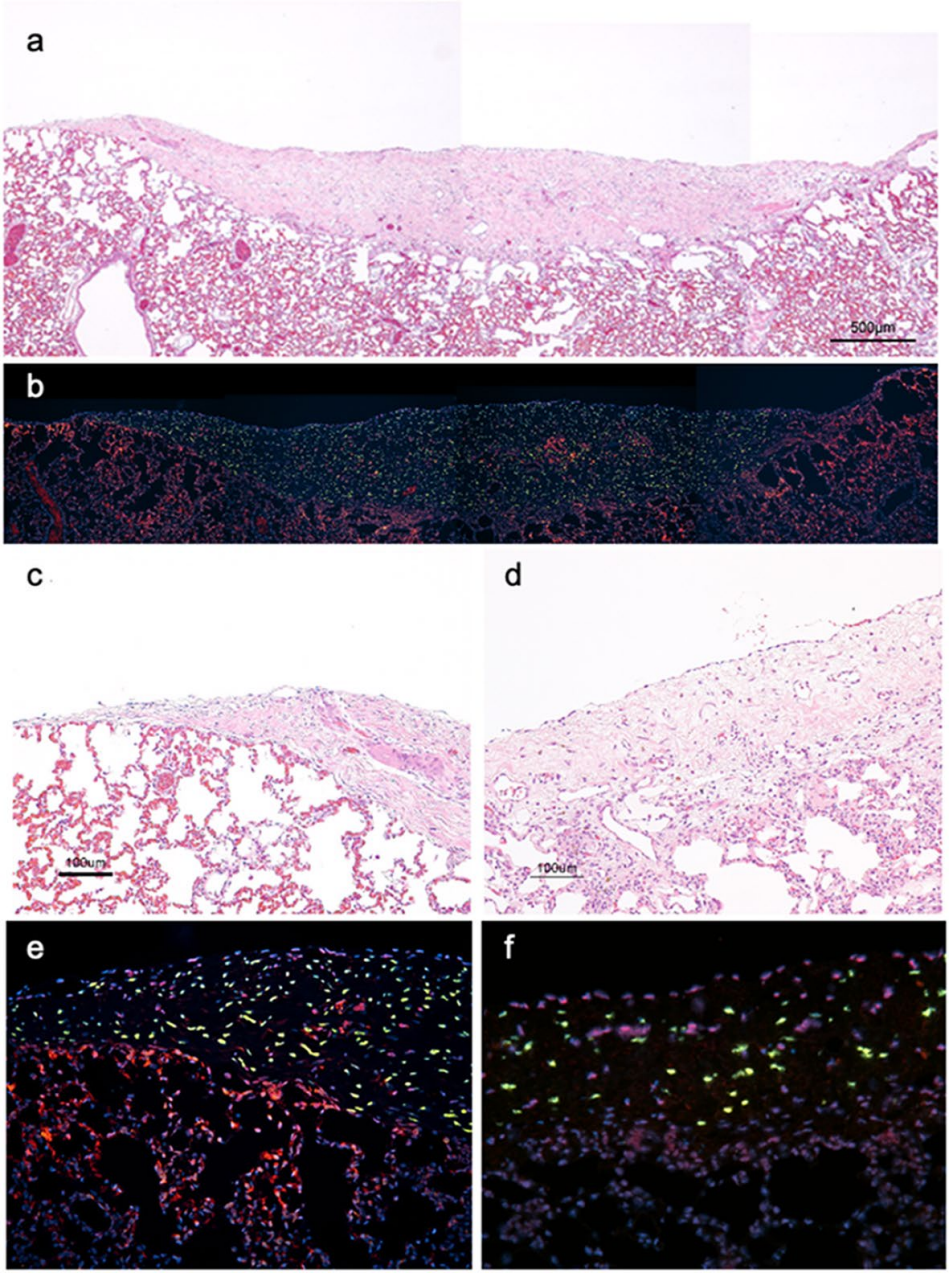
Hematoxylin and eosin (HE) staining and FISH analyses of dermal fibroblast sheet (DFS). (**a**), (**b**), (**c**), and (**e**) show representative images of rat lung DFS transplant sites 4 weeks after transplantation and (**d**) and (**f**) show representative images of human DFS transplant sites 3 months after transplantation. (**a**) and (**b**) are corresponding but combined images. (**a**) and (**c**). The human DFS is distinguished from rat pleural defect tissue, covers the pleural defect, and blood vessels are observed within the transplanted DFS. (**b**). FISH supports human fibroblast (stained green) retention within transplanted DFS, while host cells (stained red) migrate into the DFS to form new blood vessels. (**d**). Three months after DFS transplantation, rat mesothelial cells migrate to cover the surface of transplanted DFS, while blood vessels penetrate into the DFS. No signs of pulmonary fibrosis or tumor formation are observed in the host lung sites. (**e**) Four weeks after DFS transplantation, human fibroblasts remained in the DFS and whereas host cells invaded the sheet to form new blood vessels. Image is taken at a magnification of 200. (**f**). Three months after DFS transplantation, human fibroblasts surround by newly deposited extracellular matrix(ECM) declined over time in the transplanted DFS. Image is taken at a magnification of 200

Four weeks after transplantation, human DFS is distinguished from rat pleural defect tissue (Fig. 4a and c): nuclei of rat and human cells stained red and green, respectively. Both nuclei were also counterstained with DAPI (blue), revealing that human fibroblasts remained in the sheet, whereas host cells invaded the sheet to form new blood vessels (Fig. 4b and e). Combined images are shown in Fig. 4a and b. At 3 months post-transplantation, human fibroblasts remain confined to the DFS, while host mesothelial cells migrated to cover the transplanted DFS, and new blood vessels are observed within the DFS (Fig. 4d). In the transplanted DFS, human fibroblasts surrounded by newly deposited extracellular matrix(ECM) declined over time (Fig. 4f).

## Discussion

Previously, we have described the use of temperature-responsive culture dishes for the creation of transplantable sheets composed of living cells [9–12]. By a cell sheet engineering technology, we reported the ability of cell sheets to close air leaks in a dynamic function by responding to extensive expansion and contraction cycles during respiration. We had showed that DFS which can be readily cultured in the laboratory, can be effectively used as novel air leak sealant that becomes integrated with the host lung and had reported the first success of intraoperative pulmonary air leaks closure using autologous DFS in human clinical study [8].

Mesenchymal-derived fibroblasts are the most common connective tissue cells [1]. Fibroblasts are involved in both repair and regeneration of human tissues and organs, and are rapidly activated during tissue damage. This prompts them begin to proliferate, migrate to tissue damage sites, and secrete ECM as a repair event [1, 2]. Fibroblasts play an important role in epithelial-mesenchymal interactions and secrete a variety of growth factors and cytokines for paracrine signaling. All fibroblasts have similar morphology at different anatomical sites, but are known to site-specifically synthesize ECM proteins and cytokines [2, 3].

Fibroblasts are known to be involved in the development of pulmonary fibrosis [4, 5]. Unresolved inflammation, which leads to the influx of inflammatory cells into the interstitial and alveolar airspaces, damaging lung tissue with loss of normal structure. As a result of inflammation and epithelial damage, interstitial pulmonary fibroblasts that are normally present in lung connective tissue spaces become activated. These pulmonary fibroblasts then regulate and control other cellular processes that result in a profibrotic microenvironment in damaged lung tissue. Generally, when lung alveoli are damaged, fibroblasts that repair the wound produce ECM (e.g. collagen III then I) and repair the wound. However, in pulmonary fibrosis, the alveoli are repeatedly damaged for extended periods of time, and due to an excessive repair reaction, fibroblasts secrete excessive collagen thickening and hardening the alveoli walls [4, 5]. The use of fibroblast as a cell source for pleural substitutes can not be ruled out as causing pulmonary fibrosis. Against this background, in this study, we investigated whether fibroblasts engrafted after transplantation enter the lungs of the host. Compared with existing fibroblasts that cause pulmonary fibrosis, the behavior of transplanted fibroblasts is different, and it is thought that they are less likely to cause pulmonary fibrosis.

Previously, we have demonstrated that cell sheets were prepared from lung and dermal fibroblasts, respectively, and fibroblast sheets could also be effectively used as air leak sealants in the lung [6–8, 13]. In the present study, four weeks after DFS transplantation, transplanted human fibroblasts were confined in sheets, surrounded by abundant ECM produced by the transplanted fibroblast. Under tissue injury, both inflammation and repair are involved in the recruitment, activation, apoptosis, and final clearance of major effector cells [5, 14]. Among the effector cells, fibroblasts, especially myofibroblasts expressing, α-smooth muscle actin, are important [15–17]. Tissue repair events can be divided into exudative, proliferative, and extracellular matrix remodeling phases. Above all, once fibroblasts become activated in a fibroblast proliferation phase, they transform into α-smooth muscle actin–expressing myofibroblasts that secrete ECM components. The proliferative stage seeks to reduce the area of tissue injury by myofibroblasts contraction and fibroplasia [18, 19]. Fibroblasts including myofibroblasts produce and replace ECM molecules that regulate tissue strength and resilience. However, expressive ECM deposition result in fibrosis and scarring. Myofibroblasts play an important role not only in the physiological reconstruction of connective tissue after injury, but also in promoting pathological tissue deformities that characterize fibrosis [20, 21]. There were no pleurodesis in this study. Thoracic surgeons often use polyglycolic acid (PGA) sheets to repair pleuropulmonary injuries. Although PGA sheets always cause pleural adhesion, the reason why pleural injury repair with DFSs does not result in pleurodesis may be due to immunodeficient rats or because DFS does not induce inflammation.

In this study, FISH revealed human fibroblasts remained inside the sheet. On the other hand, host cells invaded the sheet to form new blood vessels. Furthermore, host mesothelial cells migrated to cover the transplanted fibroblast sheet at 3 months after transplantation. The DFS transplanted into the pleural defect produced abundant ECM by the fibroblasts inside the sheet, and although the cell number, mostly consider to be fibroblasts, had been reduced by apoptosis in the cell sheet. Then compared to autologous DFS, transplanted allogeneic DFS is less likely to engraft and likely generates less extracellular matrix to be rejected by the immune response. Transplanted fibroblasts do not invade the lungs of the host, and engrafted fibroblasts are unlikely to induce pulmonary fibrosis.

## Limitations

To the best of our knowledge, this is the first time of xenogeneic human fibroblast sheets transplanted to repair lung pleural defects using dual-color FISH analyses.

The results of this study should be interpreted in light of limitations. First, although FISH analyses revealed that even at both a and 3 months after transplantation, the transplanted human fibroblasts still remained in the sheet, important limitations of this study include its short duration and small sample size. Second, transplantations of xenogeneic human fibroblast sheets were performed using immunodeficient athymic rats. We were not able to examine the viability of cells within the cell sheets and the development of transplant-associated cancers. Therefore, further investigation with longer time periods and larger sample sizes is warranted.

On the other hand, fibroblasts are known to be involved in the development of pulmonary fibrosis. Therefore, using fibroblasts as a cell source for pleural substitutes cannot exclude the possibility of causing pulmonary fibrosis. Against this background, in the present study, we investigated whether engrafted fibroblasts enter the lungs of the host after transplantation. This study has shown that transplanted skin fibroblasts behave differently and are less likely to cause pulmonary fibrosis than existing pulmonary fibroblasts, which cause pulmonary fibrosis. The procedure of closing PALs by using a DFS as a substitute pleura, which we have reported, is thought to be one of the useful methods.

In conclusions, human DFSs transplanted onto athymic rat lung surfaces effectively seal PALs in a surgical model. Dual-color FISH analyses of DFS-transplanted human fibroblasts showed that they are retained within cell sheets and do not invade the lungs of the host.

## Data Availability

The data that support the findings of this study are available from the corresponding author upon reasonable request.

## Abbreviations

ECM: extracellular matrix
DFSs: dermal fibroblast sheet
PAL: pulmonary air leak
FISH: Fluorescence in situ hybridization
DMEM: Dulbecco’s modified Eagle’s medium
EDTA: ethylenediamine tetraacetic acid
HE: Hematoxylin and eosin
DAPI: 4,6-diamidino-2-phenylindole
CCD: charge coupled device
